# Prognosis prediction and risk factors for triple‐negative breast cancer patients with brain metastasis: A population‐based study

**DOI:** 10.1002/cam4.5575

**Published:** 2023-01-11

**Authors:** Yuqin Yang, Liguo Zhang, Wenjing Tian, Yijie Li, Qi Qin, Yinyan Mao, Xiuling Liu, Jiawei Hong, Lingzhi Hu, Qing’an Zeng, Qingling Zhang, Hong Zhao

**Affiliations:** ^1^ Department of Pathology School of Basic Medical Science, Southern Medical University Guangzhou People's Republic of China; ^2^ The Cancer Center of The Fifth Affiliated Hospital of Sun Yat‐Sen University Zhuhai People's Republic of China; ^3^ Department of Pathology Guangdong Provincial People's Hospital, Guangdong Academy of Medical Sciences Guangzhou People's Republic of China; ^4^ Guangdong Provincial Key Laboratory of Biomedical Imaging The Fifth Affiliated Hospital, Sun Yat‐sen University Zhuhai People's Republic of China; ^5^ Department of Thyroid & Breast Surgery The Fifth Affiliated Hospital, Sun Yat‐sen University Zhuhai China; ^6^ Department of Medical Oncology The Second Affiliated Hospital of Hainan Medical University Haikou People's Republic of China

**Keywords:** brain metastasis, prognosis, risk factors, triple‐negative breast cancer

## Abstract

**Background:**

Brain metastasis (BM) in triple‐negative breast cancer (TNBC) patients is associated with significant morbidity and mortality. In this research we aimed to develop a nomogram to predict the prognosis of TNBC patients with BMs (TNBC‐BM) and explore the potential risk factors.

**Methods:**

We used data from the Surveillance, Epidemiology, and End Results (SEER) database. A prognostic nomogram was built and validated based on patients with BM at newly diagnosed TNBC (nTNBC‐BM). Its effect on TNBC patients with BM was also validated in an extended group. The prognostic effect of treatment and risk factors for nTNBC‐BM were further tested.

**Results:**

A nomogram was constructed and validated to predict overall survival (OS) in TNBC‐BM patients. For patients with BM diagnosed at the initial treatment or later course, the C‐index (0.707, 0.801, and 0.685 in the training, validation, and extended groups, respectively) and calibration plots showed the acceptable prognostic accuracy and clinical applicability of the model. Surgery on the primary tumor and chemotherapy were found to confer significantly better OS (11 months vs. 4 months; 5 months vs. 3 months, respectively). In addition, advanced tumor/nodal stage and bilateral cancer were associated with a higher risk of nTNBC‐BM.

**Conclusion:**

We developed a sensitive and discriminative nomogram to predict OS in TNBC‐BM patients, both at initial diagnosis and the latter course. nTNBC‐BM patients may benefit more from surgery and chemotherapy than from radiotherapy. In addition, in the predictive model, TNBC patients harboring advanced tumor/nodal stages and bilateral tumors were more likely to have BM at initial diagnosis.

## Graphical abstract

A prognostic nomogram was developed for patients with brain metastasis at newly diagnosed triple‐negative breast cancer or latter disease course. In addition, risk factors for triple‐negative breast cancer patients with brain metastasis in initial diagnosis were also explored in present study.

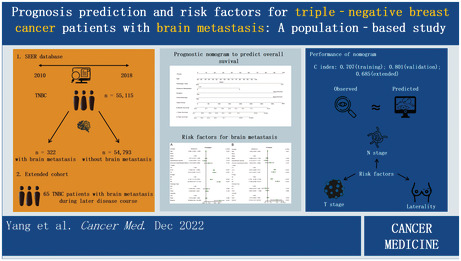

## 1 INTRODUCTION

Triple‐negative breast cancer (TNBC) is a subtype of breast cancer (BC) defined by the lack of the estrogen receptor (ER), progesterone receptor (PR), and human epidermal growth factor‐2 (HER2).^1^ Since TNBC is highly aggressive, TNBC patients exhibit a higher risk of early relapse and mortality.^2^, ^3^ Almost half of TNBC patients will develop distant metastasis during the disease course, and the median survival time is only 13.3 months after metastasis.^4^

The brain is one of the most common sites of BC metastasis and the first site in 12% of metastatic patients.^5^, ^6^ Regarding subtype, patients with TNBC have a higher risk and earlier diagnosis of BM than those with the luminal subtype of BC.^6^, ^7^ Although the lifespan of BC patients has been greatly extended because of treatment improvements, few BC patients, especially TNBC patients, with BM live longer than 1 year.^8^ In a retrospective study including 1256 BC patients diagnosed with BM, the median survival time was 8.7 months, and patients with TNBC had the worst OS at only 4.9 months.^9^

Prognostic and risk factors for BM have been identified in a BC scenario. Clinicopathological parameters, such as age, BC subtype, presence/degree of extracranial metastases, number of metastatic sites, surgical resection, and chemotherapy, were found to be related to the prognosis of BCBM.^5^, ^6^, ^8^, ^9^, ^10^, ^11^ In terms of risk factors for BCBM, younger age, poorly differentiated tumors, ER/PR‐negative status, and nodal involvement were confirmed to be associated with an increased BM risk.^8^, ^12^, ^13^ However, as TNBC has distinct characteristics, clinical factors associated with the prognosis or risk of BM in TNBC may be different from those of other subtypes. Therefore, the results based on all subtypes of BC (including a relatively low percentage of TNBC cases) may lead to serious bias when applied to patients with TNBC. Hence, to enhance the sensitivity/specificity of the clinical model and guide the treatment strategy for BM in TNBC patients, it is imperative to conduct studies and identify the clinical factors associated with the prognosis and risk by enrolling patients of this subtype exclusively, especially with a large cohort.

In the current study, a prognostic nomogram predicting OS at 6 months, 1 year, and 2 years was constructed and validated based on nTNBC‐BM patients from the SEER database. Patients with BM were enrolled to identify the long‐term prognostic effect of the model. In addition, variables associated with the risk of BM among patients were filtered out. Our present study will help further support the screening of BM patients at initial diagnosis and later disease course and predicting the prognosis of TNBC‐BM patients, and thus may aid in supporting clinical decision‐making and stratifying patients for further treatment.

## 2 METHODS

### 2.1 Study population

The clinicopathologic records of TNBC patients from the SEER database were extracted via SEER*Stat software, and treatment data were obtained by further application. The inclusion criteria included (1) diagnosed with breast cancer between 2010 and 2018; (2) pathological certification of negativity in HR and HER2; (3) complete follow‐up information; and (4) reporting source was neither autopsy nor death certificate only. The exclusion criteria were as follows: (1) age less than 18 or more than 99 years; (2) tumors in situ (Tis) or undetected (T0); and (3) unknown race, laterality or metastasis status/site. The process of patient enrollment is shown in Figure S1.

A total of 55,115 TNBC patients from the SEER database were enrolled in this study, of whom 322 patients were confirmed to have TNBC‐BM at initial diagnosis. Among the 322 patients, 249 diagnosed between 2010 and 2016 were assigned to the training cohort and used to construct the prognostic nomogram. The 73 cases diagnosed between 2017 and 2018 were used as an independent cohort to validate the prognostic model. All 55,115 patients were enrolled to determine the risk factors for BM at the initial diagnosis of TNBC.

Sixty‐five TNBC patients with BM were enrolled as an extended cohort between April 2010 and September 2017 from the Fifth Affiliated Hospital of Sun Yat‐Sen University. The inclusion criteria were as follows: (1) patients with pathologically confirmed TNBC aged between 18 and 99 years; (2) patients with complete medical records and clear follow‐up information; and (3) patients with no other history of a malignant tumor.

This study obeyed the Declaration of Helsinki and was approved by the Ethics Committee of the Fifth Affiliated Hospital of Sun Yat‐Sen University. Informed consent was obtained from each patient in the extended group.

### 2.2 Selection of variables

The following clinicopathological parameters were selected: age at diagnosis, race, T/N stage, laterality, histologic type, surgery (on the primary tumor), radiation (on the primary breast tumor), chemotherapy, and survival months. Specifically, for the extended group, survival months were defined as the period between the time of diagnosis of BM and death, and the age for analysis was the age at BM. Chemotherapy/radiation after BM, not the initial diagnosis of TNBC, was included in our study.

Age was analyzed as a continuous variable with original data. Race was divided into white, black, Asian/Pacific Islander, or American Indian/Alaska Native. The tumor stage was divided into I/II, III/IV, and Tx. For the N stage, all cases were classified into three groups: N−, N+, and Nx. Laterality was grouped into left, right, and bilateral. Histological type was grouped as invasive ductal carcinoma (IDC), invasive lobular carcinoma (ILC), and others (including micropapillary carcinoma, inflammatory carcinoma, metaplastic carcinoma and adenosquamous carcinoma). Surgery, chemotherapy and radiation were grouped as “yes” or “no/unknown.”

### 2.3 Statistical analysis

In the current study, Cox regression analyses were used to identify variables for survival in TNBC‐BM patients (presented as hazard ratios [HRs] with 95% confidence intervals [CIs]). A nomogram predicting the probability of OS at 6 months, 1 year, and 2 years was constructed based on the results of Cox regression analyses, and its predictive performance was assessed by the C‐index and calibration curves. The prognostic effect of treatment in TNBC‐BM patients was visualized using Kaplan–Meier curves and log‐rank tests. To analyze the risk variables for TNBC‐BM patients, univariate and multivariate logistic regression analyses were used (presented as odds ratios [ORs] with 95% CIs) and are shown in forest plots. All statistical analyses were performed via R software. Statistical differences were considered significant at a two‐sided *p* < 0.05.

## 3 RESULTS

### 3.1 Population characteristics

In our study, a total of 55,115 TNBC patients from the SEER database were included. Among them, at initial diagnosis of TNBC, 322 (0.6%) had BM, and the remaining 54,793 (99.4%) patients did not. All 55,115 TNBC patients were included in the logistic analysis to determine the risk factors for nTNBC‐BM. In addition, to determine whether our nomogram is able to predict the prognosis of TNBC patients who develop BM later, 65 patients were enrolled as the extended cohort. The detailed population characteristics are shown in Table S1.

### 3.2 Prognostic analysis of TNBC patients with BM at initial diagnosis

**FIGURE 1:**
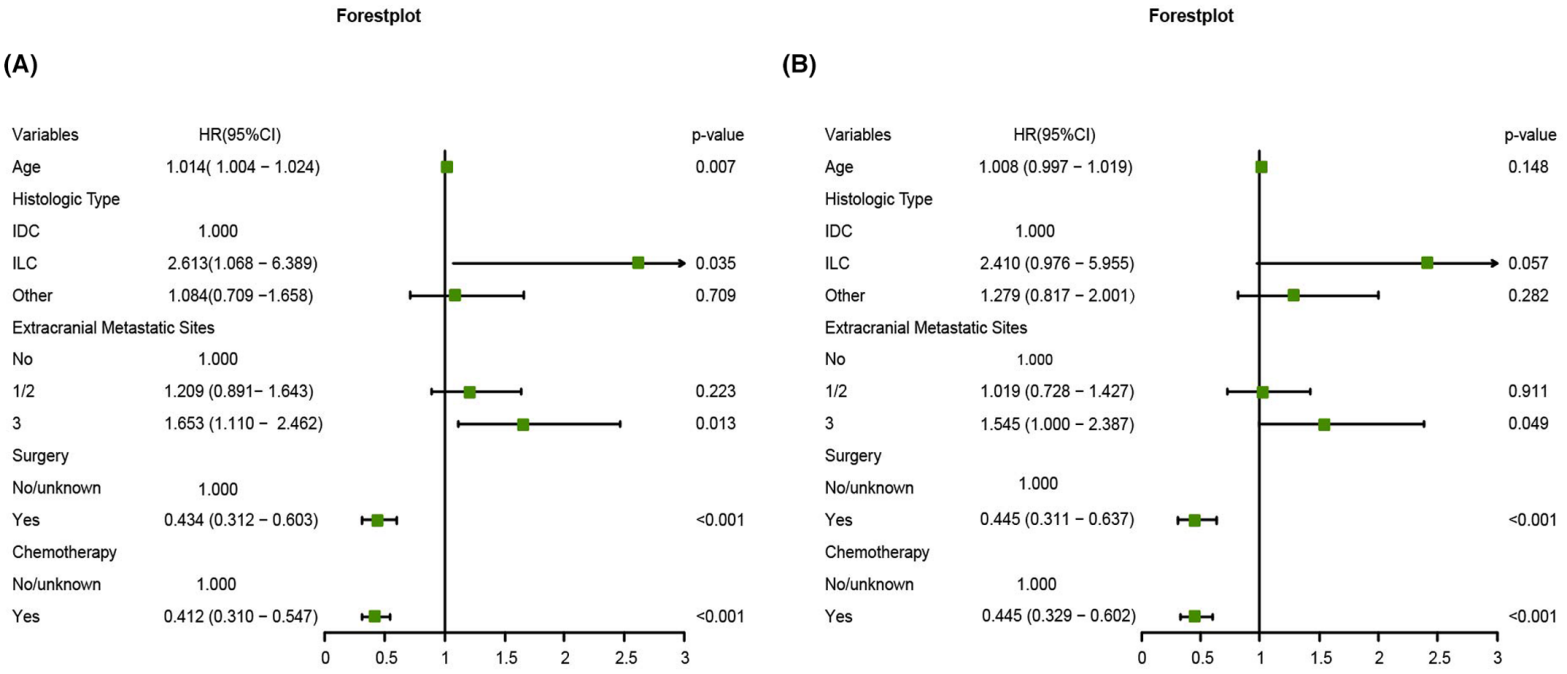
Forest plot for the potential prognostic factors for overall survival in triple‐negative breast cancer patients with brain metastasis at diagnosis by (A) univariate cox analysis and (B) multivariate cox analysis. (IDC: invasive ductal carcinoma, ILC: invasive lobular carcinoma)

Potential prognostic variables in nTNBC‐BM patients were investigated by Cox regression analysis. Older age, ILC, and three extracranial metastatic sites were found to be significantly correlated with poorer OS (HR: 1.014, 95% CI: 1.004–1.024, *p =* 0.007; HR: 2.613, 95% CI: 1.068–6.389, *p* = 0.035; HR: 1.653, 95% CI: 1.110–2.462, *p* = 0.013, respectively). In terms of treatment, nTNBC‐BM patients who received surgery or chemotherapy (HR: 0.434, 95% CI: 0.312–0.603, *p* < 0.001; HR: 0.412, 95% CI: 0.310–0.547, *p* < 0.001, respectively) had a tendency to have a significantly extended survival duration, but no significant association was found between patients who received radiotherapy and those who did not (HR: 0.776, 95% CI: 0.592–1.018, *p =* 0.067) (Figure 1A). The significant variables above were used to further analyze the association between factors and the prognosis of TNBC patients with BM. The number of extracranial metastatic sites, surgery and chemotherapy (HR: 1.545, 95% CI: 1.000–2.387, *p =* 0.049; HR: 0.445, 95% CI: 0.311–0.637, *p* < 0.001; HR: 0.445, 95% CI: 0.329–0.602, *p* < 0.001, respectively) were further confirmed to be independent risk factors for nTNBC‐BM patients (Figure 1B). The detailed results are shown in Table S2.

**FIGURE 2:**
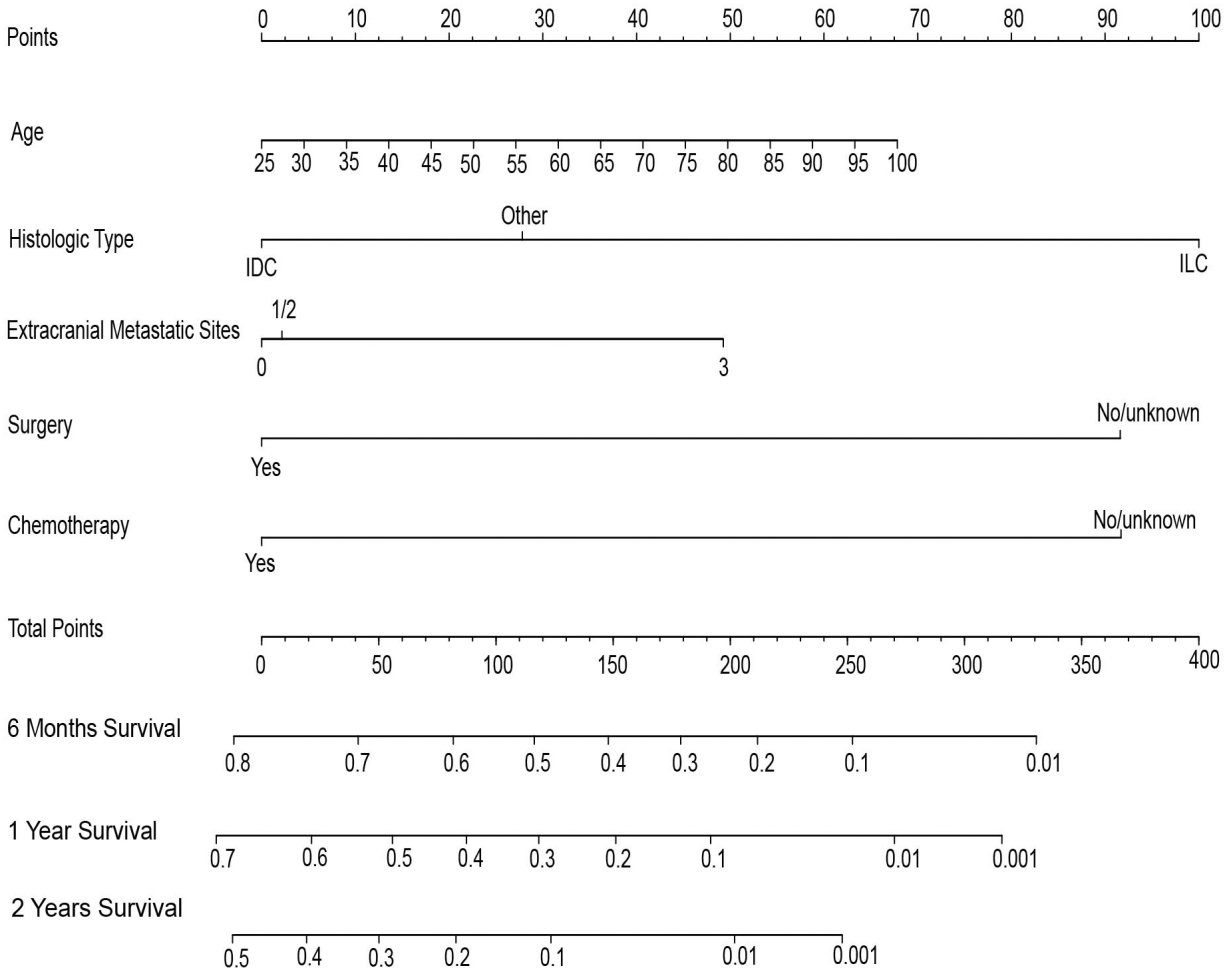
Nomograms for predicting 6‐month, 1‐year, and 2‐year overall survival of triple‐negative breast cancer patients with brain metastasis at diagnosis.

A prognostic nomogram incorporating age, histologic type, number of extracranial metastatic sites, surgery and chemotherapy was developed to predict survival in nTNBC‐BM patients based on the results above (Figure 2). By summarizing the specific points of all predictors and then measuring the total points of OS at 6 months, 1 year, and 2 years, the OS probabilities were calculated.

### 3.3 Calibration and validation of the nomogram to predict prognosis

**FIGURE 3:**
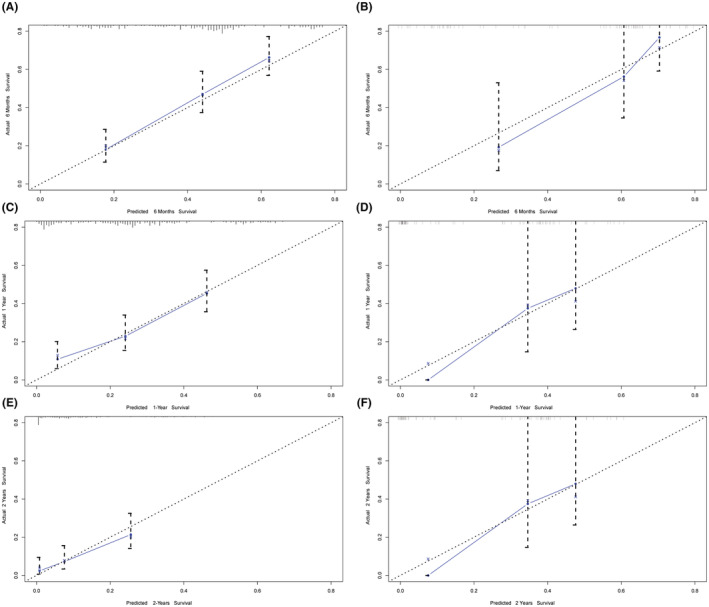
The calibration curve for predicting patient survival at (A) 6 months, (C) 1 year, and (E) 2 years in the training cohort and at (B, D, F) 6 months, 1 year, and 2 years in the validation cohort. Nomogram‐predicted probability of overall survival is plotted on the *x*‐axis; actual overall survival is plotted on the *y*‐axis.

To test the discriminative performance of this prognostic model, the C‐index and calibration curve were used in the training group and validation group. The C‐index of the nomogram was 0.707 (95% CI: 0.669–0.744). As presented by the calibration curves for the probability of survival at 6 months, 1 year, and 2 years visually, the predictive OS probability for the nTNBC‐BM patients was identical to the actual outcome (Figure 3A, C, E). In the validation group, the C‐index of the nomogram for predicting OS was 0.801 (95% CI, 0.740–0.862), and excellent agreement between predictive and observed outcomes at 6 months, 1 year, and 2 years was shown by calibration curves (Figure 3B, D, F). These results demonstrated the applicability of this clinical model in predicting OS in nTNBC‐BM patients.

### 3.4 Validation of the nomogram to predict prognosis in BM later

**FIGURE 4:**
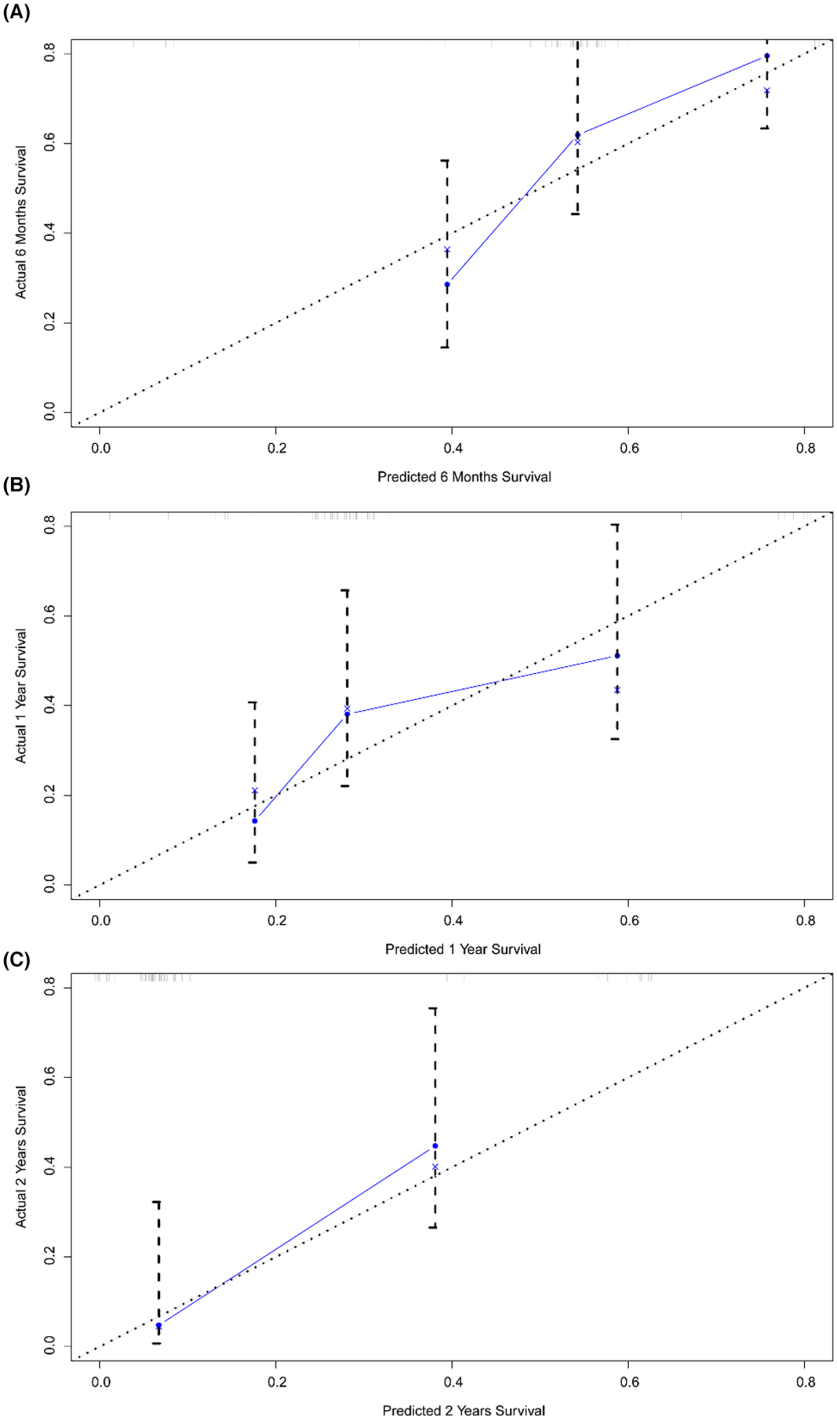
The calibration curve for predicting patient survival at (A) 6 months, (B) 1 year, and (C) 2 years in the extended cohort (TNBC patients with BM during latter disease course).

To extend the application of our model and explore whether the model is able to predict the prognosis of TNBC patients who later developed BMs, 65 patients were incorporated in our study, and the C‐index and calibration curve were generated. The nomogram showed its acceptable prognostic accuracy and clinical applicability as indicated by a C index of 0.685 (95% CI: 0.598–0.772), and the favorable consistency between the nomogram predictions and the actual observed outcomes of the 6‐month, 1‐year, and 2‐year OS were also shown by calibration curves (Figure 4A, B, C).

### 3.5 Survival analysis of treatment in TNBC patients with BM at initial diagnosis

**FIGURE 5:**
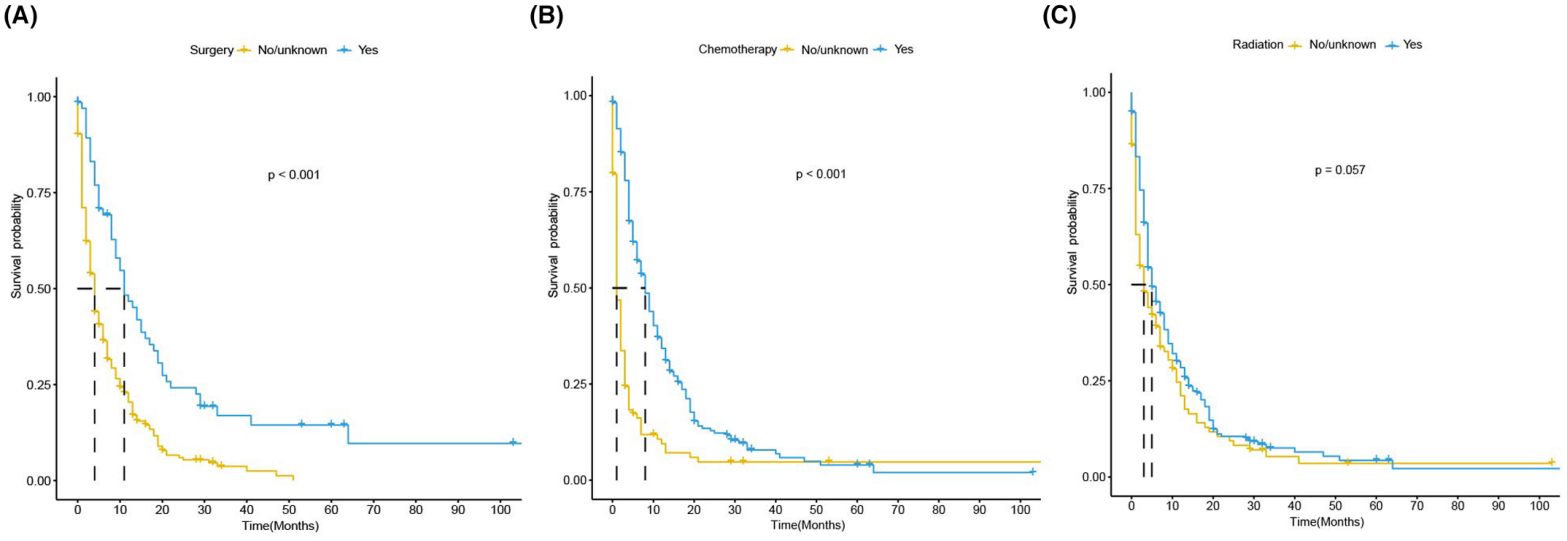
Kaplan–Meier survival curve for overall survival in triple‐negative breast cancer patients with brain metastasis at diagnosis, stratified by (A) surgery, (B) chemotherapy, (C) radiotherapy.

Survival curves were plotted to show the prognostic efficiency of factors associated with treatments directly based on 322 BM patients by the Kaplan–Meier method and log‐rank test. We found that patients who underwent surgery on the primary tumor (median OS: 11 months vs. 4 months) or systemic chemotherapy (median OS: 5 months vs. 3 months) exhibited better OS. However, patients who received radiotherapy showed no significant difference in OS (Figure 5A, B, C).

### 3.6 Risk analysis of BM in patients with newly diagnosed TNBC

After exploring the potential factors associated with OS in nTNBC‐BM patients, logistic regression was used to identify significant risk variables for BM in all 55,115 TNBC patients at the initial diagnosis. Because of its easy clinical availability and better application, even in developing countries that are unable to undergo systematic scans (such as CT and MRI), only age at diagnosis, race, T stage, N stage, laterality and histologic type were included in the analysis (Table S3).

**FIGURE 6:**
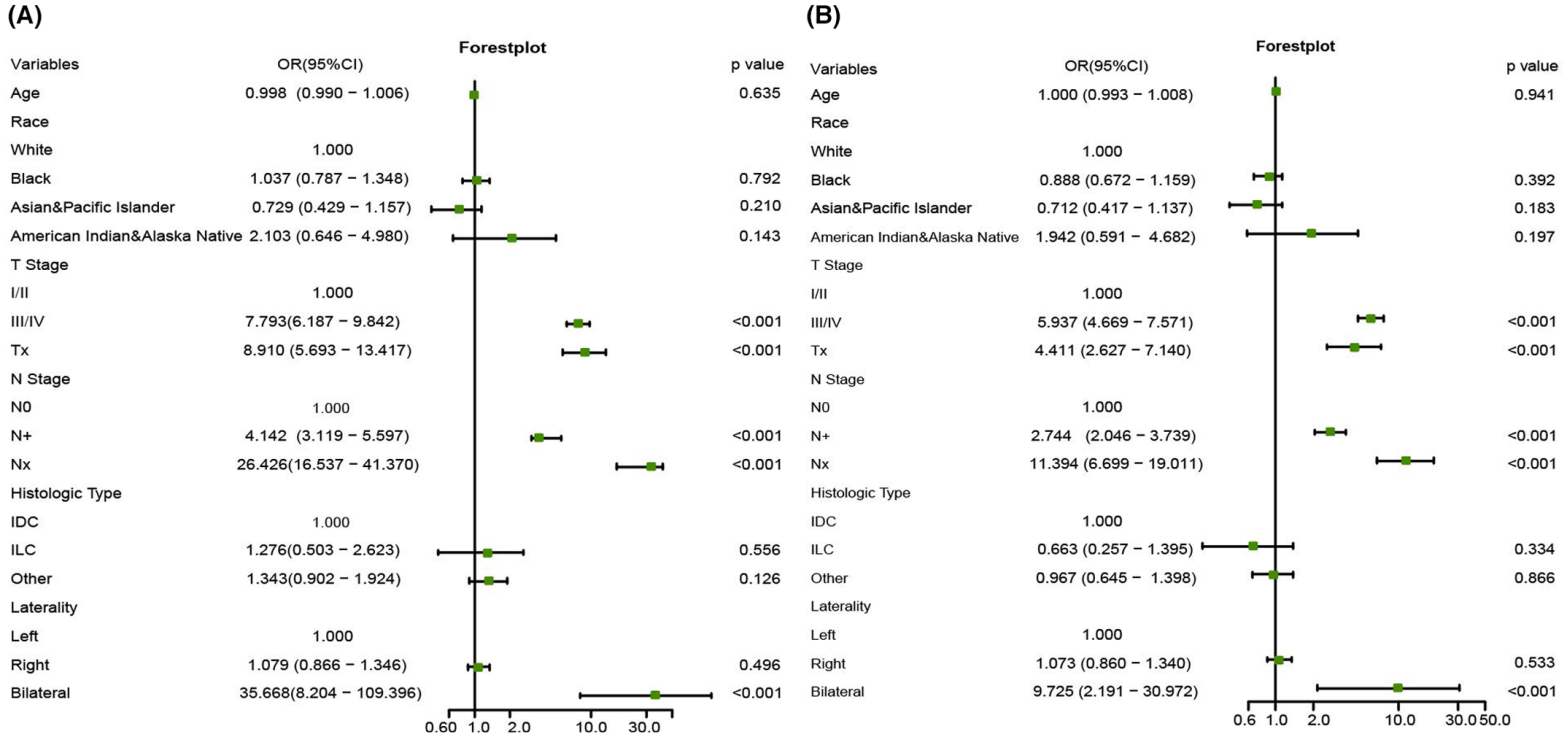
Forest plot for the potential risk factors for brain metastasis in patients with newly diagnosed triple‐negative breast cancer by (A) univariate logistic analysis and (B) multivariate logistic analysis.

Univariate analysis revealed that a more advanced T (T III/IV: OR: 7.793, 95% CI: 6.187–9.842, *p* < 0.001) or N (N+: OR: 4.142, 95% CI: 3.119–5.597, *p* < 0.001) stage was significantly related to a higher risk of BM at initial diagnosis. Compared to unilateral tumors, regardless of whether the tumor was left or right, tumors diagnosed on both sides (bilateral: OR: 35.668, 95% CI: 8.204–109.396, *p* < 0.001) were significantly more likely to develop BMs (Figure 6A). All of the factors were included in the multivariate logistic analysis. The results of the multivariate logistic regression analysis showed that a more advanced T stage (T III/IV: OR: 5.937, 95% CI: 4.669–7.571, *p* < 0.001), nodal involvement (N+: OR: 2.744, 95% CI: 2.046–3.739, *p* < 0.001), and bilateral tumors (bilateral: OR: 9.725, 95% CI: 2.191–30.972, *p* < 0.001) were still strongly associated with nTNBC‐BM (Figure 6B).

## 4 DISCUSSION

Due to the dismal clinical outcome for BC patients, especially TNBC patients with BM, clinical trials and experimental studies have been conducted to determine the risk factors or mechanisms associated with the development, progression and metastasis of BC and the prognosis of BC patients with BM.^8^, ^9^, ^10^, ^11^, ^12^, ^13^, ^14^, ^15^ However, studies to predict the survival of BC patients with BM as well as the risk of BM based on clinicopathological parameters of a large population and TNBC patients have rarely been described. In the present study, a model was trained to predict OS at 6 months, 1 year, and 2 years based on 249 nTNBC‐BM patients from 2010 to 2016, and 73 nTNBC‐BM patients between 2017 and 2018 were used as the validation cohort. Moreover, 65 TNBC patients who later developed BMs were also enrolled to study the extended application of our nomogram in BM. Moreover, based on 55,115 TNBC patients from the SEER database, logistic regression was used to explore variables associated with the risk of BM in TNBC patients at initial diagnosis.

Nomograms are accurate and helpful clinical models that can assist clinicians in predicting the possibility of a clinical outcome and have been constructed to predict the therapeutic response, recurrence/metastasis, and prognosis in patients with malignancy. Here, we developed a prognostic nomogram to predict the OS of TNBC‐BM patients. To test the discriminative performance of our nomogram, the C‐index and calibration plots were utilized. The C‐index of our model was 0.707 (95% CI: 0.669–0.744) in the training group and 0.801 (95% CI, 0.740–0.862) in the validation group. According to previous studies, a nomogram with a C‐index >0.7 was regarded as having an acceptable sensitivity and specificity.^16^ Moreover, calibration curves showed good agreement in both cohorts, which suggested the reliability of the model. In the extended group, the C‐index of our prognostic nomogram was 0.685 (95% CI: 0.598–0.772), which was only slightly <0.7 and may be the result of a relatively low sample size, and favorable consistency was also shown by the calibration curves. Thus, the nomogram constructed in the present study was proven to have an excellent discriminative ability in predicting the prognosis of TNBC‐BM patients, both diagnosed at initial treatment and the latter course, by incorporating clinicopathological characteristics. We hope that it can assist clinicians in identifying TNBC‐BM patients with a higher risk of mortality and aid in treatment strategy making.

In the present study, clinicopathological factors, including age, histologic type, number of extracranial metastatic sites, surgery, and chemotherapy, were related to OS in nTNBC‐BM patients in univariate Cox regression analysis; of these factors, the number of extracranial metastatic sites, surgery, and chemotherapy were independent prognostic factors. Older age has been found to be correlated with poorer prognosis in BC patients with BM by some researchers.^5^, ^10^, ^12^, ^13^ A study by Subbiah found that younger patients tended to have fewer complications and better Karnofsky performance scores, which allowed younger patients to receive significantly more systemic treatment at the time of BM.^17^ Epidemiological results have shown that most TNBC patients are diagnosed at a younger age; however, they have the worst prognosis after developing BM.^8^, ^9^, ^18^ Therefore, it is reasonable to speculate that the highly aggressive behavior of TNBC outweighs the benefit of a TNBC diagnosis at a young age. ILCs were another factor associated with shorter OS in nTNBC‐BM patients. There is an overall consensus that ILCs respond poorly to chemotherapeutic drugs, which is the major systemic treatment for TNBC.^19^, ^20^ A study by Cocquyt et al. indicated that the overall response for ILCs was 50%, compared to 75% for IDCs. Moreover, the chemotherapy regimen used was not found to affect the results.^21^ The number of extracranial metastatic sites was found to be an independent prognostic factor. Liu et al noted that patients with more than 1 metastatic organ site tended to have a remarkably poorer OS by analyzing 1517 BC patients with BM at initial diagnosis, and this association remained valid after adjusting for confounding variables.^10^

Regarding treatment variables (surgery, chemotherapy, and radiotherapy), our results showed that palliative surgery and systemic chemotherapy were significantly related to the OS of nTNBC‐BM patients, but radiotherapy was not. Previously, it was believed that resection of the primary tumor in metastatic patients was palliative and performed only to relieve symptoms. Patients with metastatic BC were recognized as having lost the opportunity for surgery and received only systemic therapy.^22^ However, studies on the effect of surgery of the primary tumor on metastatic BC have shown controversial results. Some former retrospective studies suggested that patients with distant metastasis cannot benefit from palliative surgery, even though surgical resection may promote the progression of systemic metastases.^23^, ^24^ However, studies based on large populations have indicated that BC patients with BM may achieve better OS and breast cancer‐specific survival after tumor resection.^10^, ^25^ In addition, chemotherapy was significantly associated with better survival of BM patients in our study. Studies have shown that neoadjuvant chemotherapy for TNBC has a significantly higher ratio of pathological response than that for the luminal subtype and is associated with a significantly better prognosis in TNBC patients.^26^, ^27^, ^28^ Therefore, future studies should explore the appropriate chemotherapy drugs and optimize the chemotherapy regimens to ensure good treatment outcome and prognosis in TNBC patients, especially in those with organ metastasis.

By using logistic regression analysis, more advanced T stage, lymph node metastasis (LNM) and bilateral tumors strongly correlated with a higher risk of BM in TNBC patients at initial diagnosis. Tumor stage/size reflects the condition of the primary tumor, and a higher tumor stage/larger size means that the tumor harbors more aggressive behavior. Kim et al. enrolled 206,913 BC patients and identified T stage as an independent predictive cofactor for BM; as the T stage advanced, the risk of BM doubled.^29^ In addition, in studies of lung cancer, the results showed that tumor size was positively associated with the incidence of BM.^30^, ^31^ Nodal involvement was found to be another independent predictor for BM in patients with newly diagnosed TNBC. The dissemination of cancer cells to the brain was previously believed to occur hematogenously in the absence of a classical lymphatic drainage system in the central nervous system, but according to the latest research, functional lymphatic vessels were found in brain tissues and connected the lymph node.^32^, ^33^ A retrospective study by Zhu et al. included 117,442 non‐small cell lung cancer patients and demonstrated that LNM was associated with a significantly higher possibility of BM at initial diagnosis.^30^ However, compared with other subtypes, TNBC was demonstrated to have the lowest risk of LNM,^34^, ^35^ which may mean that LNM was not the major driving factor for BM in TNBC. To increase the accuracy of prediction, more variables should be included in further studies. In our analysis, laterality was also found to be associated with BM because invasive TNBC diagnosed on both sides was positively related to the risk of BM. The reason for a higher risk of BM for bilateral BC might be the larger tumor burden or the fact that multifocal locations create a larger chance of metastases.^36^ In our study, invasive TNBC patients with bilateral BC were almost 10 times (OR = 9.93) more likely to develop BM. The results indicated that, compared with other subtypes, bilateral TNBC has much more aggressive behavior.

In clinical practice, brain magnetic resonance imaging (MRI) is routinely recommended for patients with suspicious central nervous system symptoms but not for asymptomatic patients.^37^ However, asymptomatic BM patients (diagnosed by screening) were found to have better outcomes than those with central nervous system symptoms and may receive more benefits from the treatment.^38^, ^39^ Due to the relatively low availability and the high cost, the usage of routine brain MRI screening is limited. Thus, the risk factor for nTNBC‐BM we found may help to identify TNBC patients with BM at initial diagnosis and aid in clinical strategy making.

There are limitations in our study. First, this study was a retrospective study, which may lead to an unavoidable bias. Second, in the extended group, this was a relatively small group of patients from a single center, and larger cohorts are needed to validate the effect of our model to predict the prognosis of BM. Third, some variables, such as blood index and the number or location of BMs, may also be potential factors affecting the prognosis of TNBC‐BM patients and need to be incorporated into our model. However, these variables could not be included in our study because they were unavailable in the SEER database. Their prognostic effects on TNBC‐BM patients deserve further exploration. Fourth, only correlative analysis was conducted between the risk factor and BM, and experimental studies are needed to classify whether there is causality between the risk factors above and BM and the mechanism of BM in the future.

In conclusion, we are the first to construct and validate a prognostic nomogram for OS in TNBC‐BM patients, including those diagnosed at initial treatment or later in the disease course, which can help clinicians evaluate the high risk of mortality in patients and develop a more appropriate treatment strategy. In our study we also found that for patients with newly diagnosed TNBC, advanced T/N stage and bilateral tumors were positively associated with BM.

## AUTHOR CONTRIBUTIONS

**Yuqin Yang:** Formal analysis (lead); investigation (lead); methodology (lead); software (lead); visualization (equal); writing – original draft (lead). **Liguo Zhang:** Formal analysis (equal); investigation (equal); methodology (equal); software (equal); validation (equal); writing – original draft (equal). **Wenjing Tian:** Investigation (equal); methodology (equal); software (equal); writing – original draft (equal). **Yijie Li:** Investigation (equal); validation (equal); writing – original draft (equal). **Qi Qin:** Investigation (equal); methodology (equal); writing – original draft (equal). **Yinyan Mao:** Methodology (equal); software (equal). **Xiuling Liu:** Investigation (equal); methodology (equal). **Jiawei Hong:** Investigation (equal); methodology (equal). **Lingzhi Hu:** Investigation (equal); methodology (equal). **Qing'an Zeng:** Conceptualization (equal); project administration (equal); supervision (equal); validation (equal); writing – review and editing (equal). **Qingling Zhang:** Conceptualization (equal); project administration (equal); supervision (equal); validation (equal); writing – review and editing (equal). **Hong Zhao:** Conceptualization (lead); data curation (lead); formal analysis (lead); funding acquisition (lead); project administration (lead); resources (lead); supervision (lead); validation (lead); writing – review and editing (lead).

## CONFLICT OF INTEREST

There are no conflicts of interest.

## ETHICS APPROVAL AND CONSENT FOR PARTICIPATE

This study obeyed the Declaration of Helsinki and was approved by the Ethics Committee of the Fifth Affiliated Hospital of Sun Yat‐Sen University (reference number: K324‐1). Informed consent was obtained from each patient in the extended group.

## Supporting information


Appendix S1.

## Data Availability

The data analyzed in training and validation group is available at https://seer.Cancer.gov/ . The data of extended group are available on request from the corresponding author. The data are not publicly available due to privacy or ethical restrictions.
