# Supplementary material for: Prognosis prediction and risk factors for triple‐negative breast cancer patients with brain metastasis: A population‐based study
Source: Cancer Med. 2023 Jan 11;12(7):7951–61. doi: 10.1002/cam4.5575 (PMC10134296; doi:10.1002/cam4.5575)
Supplement: Supplementary file 1 — Appendix S1. [file CAM4-12-7951-s001.doc]

**Supporting Materials**

**Supporting Figure 1:** Flowchart of patient selection. Detailed selection of TNBC patients diagnosis at 2010–2018 from SEER database.


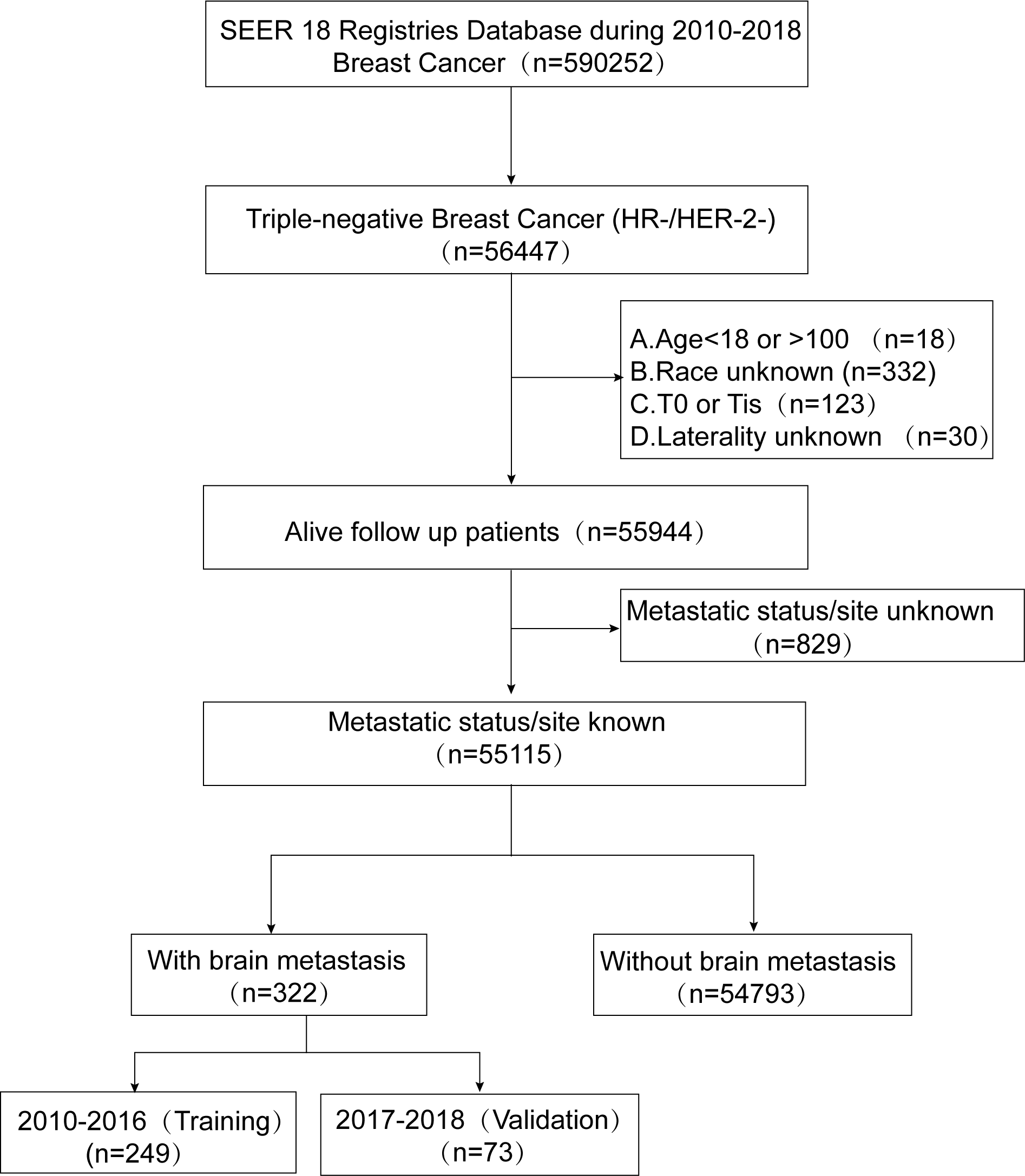


**Supporting Table 1**. Demographics and clinicopathologic characteristics of invasive triple-negative breast cancer patients.

| Variables | SEER database | | | | Patients with BM latter  (Extended group)(n=65) |
| --- | --- | --- | --- | --- | --- |
|  | All patients, (n =55115) | 2010-2016  With Brain metastasis  (Training) (n=249) | 2017-2018  With Brain metastasis  (Validation) (n=73) | Without Brain metastasis  (n=54793) |  |
| Age, median (IQR) | 59(49, 69) | 58(49, 66) | 62(54,69） | 59(49,69) | 50(44,56) |
| Race, n(%)  White  Black  Asian&Pacific Islander  American Indian&Alaska Native | 39,329(71.4)  11,498(20.8)  3,962(7.2)  326(0.6) | 180(72.3)  53(21.3)  13(5.2)  3(1.2) | 51(69.8)  17(23.3)  4(5.5)  1(1.4) | 39,098(71.3)  11,428(20.9)  3,945(7.2)  322(0.6) | All are Chinese |
| T Stage  I/II  III/IV  Tx | 45,828(83.1)  8,194(14.9)  1,093(2.0) | 100(40.2)  130(52.2)  19(7.6) | 25(34.2)  41(56.2)  7(9.6) | 45,703(83.4)  8,023(14.6)  1,067(2.0) | 50(76.9)  12(18.5)  3(4.6) |
| N Stage  N0  N+  Nx | 26,898(48.9)  27,662(50.1)  555(1.0) | 44(17.7)  180(72.3)  25(10.0) | 15(20.5)  54(74.0)  4(5.5) | 26,842(49.0)  27,425(50.1)  526(0.9) | 20(30.8)  43(66.1)  2(3.1) |
| Laterality, n(%)  Left  Right  Bilateral | 28,336(51.4)  26,761(48.6)  18(0.03) | 121(48.6)  127(51.0)  1(0.4) | 37(50.7)  34(46.6)  2(2.7) | 28,178(51.4)  26,600(48.6)  15(0.02) | 33(50.8)  28(43.1)  4(6.2) |
| Histologic Type  IDC  ILC  Other | 50,345(91.3)  829(1.5)  3,941(7.2) | 223(90.0)  5(2.0)  21(8.0) | 63(86.3)  1(1.4)  9(12.3) | 50,059((91.4)  823(1.5)  3,911(7.1) | 55((84.6)  3(4.6)  7(10.8) |
| Extracranial Metastatic Sites,n(%)  0  1/2  3 | 52,658(95.6)  2,280(4.1)  177(0.3) | 71(28.5)  135(54.2)  43(17.3) | 22(30.2)  42(57.5)  9(12.3) | 52,565(95.9)  2,103(3.9)  125(0.2) | 13(20.0)  46(70.8)  6(9.2) |
| Surgery, n(%)  No/unknown  Yes | 4,960(9.0)  50,155(91.0) | 192(77.1)  57(22.9) | 64(87.7)  9(12.3) | 4,704(8.6)  50,089(91.4) | 4(6.2)  61(94.8) |
| Radition，n(%)  No/unknown  Yes | 28,750(52.2)  26,365(47.8) | 83(33.3)  166(66.7) | 26(35.6)  47(64.4) | 28,641(52.3)  26,152(47.7) | 43(66.2)  22(33.8) |
| Chemotherapy，n(%)  No/unknown  Yes | 14,253(25.9)  40,862(74.1) | 76(30.5)  173(69.5) | 32(43.8)  41(56.2) | 14,145(25.8)  40,648(74.2) | 24(36.9)  41(63.1) |

Abbreviations: IDC: invasive ductal carcinoma; ILC: invasive lobular carcinoma; IQR: interquartile range.

**continued** Characteristics of triple-negative breast cancer patients.

**Supporting Table 2**. Univariate and multivariate cox analysis of overall survival of the training cohort.

| Variables | Univariate analysis | | |  | Multivariate analysis | | |
| --- | --- | --- | --- | --- | --- | --- | --- |
|  | *HR* | 95%Cl | *p-value* |  | *HR* | 95%Cl | *p-value* |
| age | 1.014 | 1.004 - 1.024 | 0.007 |  | 1.008 | 0.997 - 1.019 | 0.148 |
| Race  White  Black  Asian&Pacific Islander  American Indian&Alaska Native | 1.000  1.172  0.617  0.881 | 0.856 - 1.605  0.326 -1.170  0.280 - 2.765 | 0.322  0.139  0.828 |  |  |  |  |
| T Stage  I/II  III/IV  Tx | 1.000  0.981  1.427 | 0.749 - 1.285  0.859 - 2.369 | 0.891  0.169 |  |  |  |  |
| N Stage  N0  N+  Nx | 1.000  1.149  1.078 | 0.808 - 1.633  0.642 - 1.811 | 0.439  0.776 |  |  |  |  |
| Laterality  Left  Right  Bilateral | 1.000  1.102  6.819 | 0.852 - 1.541  0.935 - 49.722 | 0.459  0.058 |  |  |  |  |
| Histologic Type  IDC  ILC  Other  **continued** Cox analysis of the training cohort. | 1.000  2.613  1.084 | 1.068 - 6.389  0.709 - 1.658 | 0.035  0.709 |  | 1.000  2.410  1.279 | 0.976 - 5.955  0.817 - 2.001 | 0.057  0.282 |
| Extracranial Metastatic Sites  No  1/2  3 | 1.000  1.209  1.653 | 0.891 - 1.643  1.110 - 2.462 | 0.2233  0.013 |  | 1.000  1.019  1.545 | 0.728 - 1.427  1.000 - 2.387 | 0.911  0.049 |
| Surgery  No/unknown  Yes | 1.000  0.434 | 0.312 - 0.603 | <0.001 |  | 1.000  0.445 | 0.311 - 0.637 | <0.001 |
| Radition  No/unknown  Yes | 1.000  0.776 | 0.592 - 1.018 | 0.067 |  |  |  |  |
| Chemotherapy  No/unknown  Yes | 1.000  0.412 | 0.310 - 0.547 | <0.001 |  | 1.000  0.445 | 0.329 - 0.602 | <0.001 |

Abbreviations: IDC: invasive ductal carcinoma; ILC: invasive lobular carcinoma; IQR: interquartile range; CI: confidence interval; HR: hazard ratios.

**Supporting Table 3**. Univariate and multivariate logistic regression analysis to identify risk factors for brain metastasis in invasive triple-negative breast cancer patients at diagnosis

| Variables | Univariate analysis | | |  | Multivariate analysis | | |
| --- | --- | --- | --- | --- | --- | --- | --- |
|  | *OR* | 95%Cl | *p-value* |  | *OR* | 95%Cl | *p-value* |
| age | 0.998 | 0.990 - 1.006 | 0.635 |  | 1.000 | 0.993 - 1.008 | 0.941 |
| Race  White  Black  Asian&Pacific Islander  American Indian&Alaska Native | 1.000  1.037  0.729  2.103 | 0.787 - 1.348  0.429 - 1.157  0.646 - 4.980 | 0.792  0.210  0.143 |  | 1.000  0.888  0.712  1.942 | 0.672 - 1.159  0.417 - 1.137  0.591 - 4.682 | 0.392  0.183  0.197 |
| T stage  I/II  III/IV  Tx | 1.000  7.793  8.910 | 6.187 - 9.842  5.693 - 13.417 | <0.001  <0.001 |  | 1.000  5.937  4.411 | 4.669 - 7.571  2.627 - 7.140 | <0.001  <0.001 |
| N stage  N0  N+  Nx | 1.000  4.142  26.426 | 3.119 - 5.597  16.537 - 41.370 | <0.001  <0.001 |  | 1.000  2.744  11.394 | 2.046 -3.739  6.699 - 19.011 | <0.001  <0.001 |
| Histologic Type  IDC  ILC  Other | 1.000  1.276  1.343 | 0.503 - 2.623  0.902 - 1.924 | 0.556  0.126 |  | 1.000  0.663  0.967 | 0.257 - 1.395  0.645 - 1.398 | 0.334  0.866 |
| Laterality  Left  Right  Bilateral | **continued** Risk factors for brain metastasis.  1.000  1.079  35.668 | 0.866 - 1.346  8.204 - 109.396 | 0.496  <0.001 |  | 1.000  1.073  9.725 | 0.860 - 1.340  2.191 - 30.972 | 0.533  <0.001 |

Abbreviations: IDC: invasive ductal carcinoma; ILC: invasive lobular carcinoma; CI, confidence interval; OR, odds ratios.
